# Implicit learning seems to come naturally for children with autism, but not for children with specific language impairment: Evidence from behavioral and ERP data

**DOI:** 10.1002/aur.1954

**Published:** 2018-04-20

**Authors:** Fenny S. Zwart, Constance Th.W.M. Vissers, Roy P.C. Kessels, Joseph H.R. Maes

**Affiliations:** ^1^ Donders Institute for Brain Cognition and Behaviour, Radboud University Nijmegen The Netherlands; ^2^ Behavioural Science Institute Nijmegen The Netherlands; ^3^ Royal Dutch Kentalis Sint‐Michielsgestel The Netherlands; ^4^ Department of Medical Psychology Radboud University Medical Center Nijmegen The Netherlands; ^5^ Vincent van Gogh Institute for Psychiatry Venray The Netherlands

**Keywords:** ASD, SLI, implicit learning, SRT task, N2b, P3, ERPs

## Abstract

Autism spectrum disorder (ASD) and specific language impairment (SLI) are two neurodevelopmental disorders characterized by deficits in verbal and nonverbal communication skills. These skills are thought to develop largely through implicit—or automatic—learning mechanisms. The aim of the current paper was to investigate the role of implicit learning abilities in the atypical development of communication skills in ASD and SLI. In the current study, we investigated Response Times (RTs) and Event Related Potentials (ERPs) during implicit learning on a Serial Reaction Time (SRT) task in a group of typically developing (TD) children (*n* = 17), a group of autistic children (*n* = 16), and a group of children with SLI (*n* = 13). Findings suggest that learning in both ASD and SLI are similar to that in TD. However, electrophysiological findings suggest that autistic children seem to rely mainly on more automatic processes (as reflected by an N2b component), whereas the children with SLI seem to rely on more controlled processes (as reflected by a P3 component). The TD children appear to use a combination of both learning mechanisms. These findings suggest that clinical interventions should aim at compensating for an implicit learning deficit in children with SLI, but not in children with ASD. Future research should focus on developmental differences in implicit learning and related neural correlates in TD, ASD, and SLI. ***Autism Res*** *2018, 11: 1050–1061*. © 2018 The Authors Autism Research published by International Society for Autism Research and Wiley Periodicals, Inc.

**Lay Summary:**

Autism and Specific Language Impairment (SLI) are two disorders characterized by problems in social communication and language. Social communication and language are believed to be learned in an automatic way. This is called “implicit learning.” We have found that implicit learning is intact in autism. However, in SLI there seems different brain activity during implicit learning. Maybe children with SLI learn differently, and maybe this different learning makes it more difficult for them to learn language.

## Introduction

Social and communication skills, including language, are thought to develop largely through implicit learning processes. Implicit learning refers to incidental learning that leads to knowledge that we are not consciously aware of, and is debatably believed to be distinctively different from explicit (or conscious) learning [e.g., Abrahamse, Jiménez, Verwey, & Clegg, 2010; Reber, 1967, 2013]. We use this implicit learning system to master grammar rules of our mother tongue [e.g., Saffran, Aslin, & Newport, 1996], and to learn how to deal with our highly complex social environment [Lieberman, 2000]. It is therefore not surprising that implicit learning is often studied in developmental disorders characterized by deficits in these language and social communication skills, such as autism spectrum disorder (ASD) and specific language impairment [SLI; see Obeid, Brooks, Powers, Gillespie‐Lynch, & Lum, 2016; Zwart, Vissers, Kessels, & Maes, 2017].

At a neurobiological level, implicit and explicit learning can be dissociated, although some overlap and interactions have also been reported. Implicit learning is subserved by widespread neural networks depending on the experience at hand [as reviewed by Reber, 2013]. Implicit skill learning often involves perceptual‐motor learning and therefore engagement of the basal ganglia and the cerebellum [e.g., Janacsek, Fiser, & Németh, 2012; Krishnan, Watkins, & Bishop, 2016]. Explicit learning relies mainly on the Medial Temporal Lobe, including the hippocampus, with connections to other brain areas such as the prefrontal cortex [e.g., Reber, 2013]. However, it has been found that in some situations the hippocampus is also involved in implicit learning [e.g., Hannula & Greene, 2012; Poldrack & Rodriguez, 2003], and that the neural networks of implicit and explicit learning interact [Destrebecqz et al., 2005; Ullman, 2004]. Thus, the different neural networks involved in implicit and explicit learning can overlap and interact depending on the learning task.

Implicit learning is commonly investigated using the Serial Reaction Time (SRT) task [Nissen & Bullemer, 1987]. In this task, participants are asked to respond to visual stimuli as fast as they can by pressing a button. Unknown to the participants, these stimuli follow a repeating sequence. Often, random stimuli or blocks of random stimuli are inserted in the task, giving the task a probabilistic character (i.e., the stimuli follow the sequence only with a certain probability). Implicit learning is present if responses to sequenced stimuli are faster than those to random stimuli, despite the lack of task instruction that would have encouraged a deliberate seeking of underlying rules and participant awareness. Although the SRT task reliably evokes learning, it is challenging to guarantee the implicit nature of the learning.

The implicit learning definition entails two different aspects: the incidental nature of the learning (the process) and the unconscious nature of the knowledge [the product; e.g., Abrahamse et al., 2010; Perruchet, 2008; Reber, 1967]. In the SRT task, it is assumed that learning is incidental in nature, because there is no instruction to learn. However, it cannot be ruled out that participants have an intrinsic intention to learn. Participant awareness of the sequence (knowledge) is usually measured by a verbal interview at the end of the experiment, in which the participants are asked whether they noticed the sequence, but it can also be estimated by analyzing large drops in Response Times [RTs; “RT‐drops”; Haider & Rose, 2007]. It has been found that a substantial part of the participants show signs of (partial) awareness on the SRT task [e.g., Haider & Rose, 2007]. It is believed that awareness can be prevented by using a probabilistic sequence rather than a (simple) deterministic sequences [e.g., Jiménez, Méndez, & Cleeremans, 1996], and a probabilistic task might therefore be particularly sensitive to deficits in implicit learning.

Evidence for different neural mechanisms during incidental and intentional learning on the SRT task has been found [e.g., Ferdinand, Mecklinger, & Kray, 2008; Fletcher et al., 2004; Rüsseler, Hennighausen, Münte, & Rösler, 2003]. Studies using Event Related Potentials (ERPs) measures on the SRT Task have repeatedly found a negative deflection called the N2b component, followed by a positive deflection called the P3 component. Several studies have shown that the components are more strongly enhanced when explicit knowledge is present compared to when it is absent, and therefore concluded that both N2b and P3 may be markers of explicit learning [e.g., Eimer, Goschke, Schlaghecken, & Stürmer, 1996; Miyawaki, Sato, Yasuda, Kumano, & Kuboki, 2005; Schlaghecken, Stürmer, & Eimer, 2000]. However, Fu, Bin, Dienes, Fu, and Gao [2013] have argued that these studies may not have been sensitive enough to implicit learning, because: (a) their measures of implicit sequence knowledge may have been contaminated with sequence parts that were not learned at all; and (b) the studies may have been underpowered. By adding a more sensitive measure for implicit knowledge, the authors found that the N2b component is particularly pronounced during implicit learning, whereas the later P3 component is related to explicit learning [Fu et al., 2013], in line with similar claims by other authors [Ferdinand et al., 2008; Zwart, Vissers, Van der Meij, Kessels, & Maes, 2017]. Although the exact interpretation of the N2b and P3 can be debated, the latency of the components suggests that the early N2b component reflects lower level cognitive processes, such as the automatic detection of deviations in the environment, compared to the later P3 component, which may reflect more controlled or conscious ways of processing.

The majority of studies that used the SRT task to investigate implicit learning in ASD show intact implicit performance [Foti, De Crescenzo, Vivanti, Menghini, & Vicari, 2015; Obeid et al., 2016; Zwart, Vissers, Kessels, et al., 2017]. However, several studies find evidence for (subtle) impairments in learning on the SRT task in ASD [Gordon & Stark, 2007; Mostofsky, Goldberg, Landa, & Denckla, 2000; Sharer, Mostofsky, Pascual‐Leone, & Oberman, 2016; Travers, Kana, Klinger, Klein, & Klinger, 2015]. It has been suggested that autistic participants rely more on explicit learning than nonautistic participants, perhaps as a compensatory mechanism for a deficit in implicit learning [e.g., Klinger, Klinger, & Pohlig, 2007]. In line with this, ERP findings of our previous SRT task study suggested a greater reliance on (intrinsic) intentional learning mechanisms in autistic adults (as reflected by a P3 component) compared to nonautistic adults (who showed an enhanced N2b component), whereas behavioral performance was intact [Zwart, Vissers, van der Meij, et al., 2017]. Similarly, a recent fMRI study found different neural correlates for autistic participants compared to TD individuals, while their behavioral performance was similar [Sharer et al., 2015]. Thus, behavioral learning on the SRT task seems intact in ASD, but there is some evidence for different neural substrates, perhaps reflecting an altered underlying learning mechanism.

Implicit learning has also been studied widely in SLI, a developmental disorder characterized by language impairments in the absence of sensory, medical, or intellectual deficits [Bishop, 1992; Tomblin et al., 1997]. It has been proposed that these language impairments, in particular the grammar difficulties, result from a deficit in overall procedural (or implicit) learning [Ullman & Pierpont, 2005] or from a more specific deficit in implicit sequence learning [Hsu & Bishop, 2014]. Indeed, most studies reported impaired implicit learning on the SRT task in SLI [Lum, Conti‐Ramsden, Morgan, & Ullman, 2014; Obeid et al., 2016; Zwart, Vissers, Kessels, et al., 2017], although several studies demonstrated intact performance [e.g., Gabriel, Maillart, Guillaume, Stefaniak, & Meulemans, 2011; Lum & Bleses, 2012]. Similar to ASD, a compensatory role of explicit learning has been suggested in SLI too [Lum et al., 2014], but it is still debated whether this explicit learning is fully intact [e.g., Hsu & Bishop, 2014; Ullman & Pierpont, 2005]. Supporting this are findings that grammar abilities were associated with implicit learning in TD children, whereas they were associated with explicit learning in children with SLI [Lum et al., 2014]. To the best of our knowledge, no studies to date have investigated neural substrates of implicit learning in SLI. A recent study did show altered ERPs in subgroups of SLI during a language task [Haebig, Weber, Leonard, Deevy, & Tomblin, 2017].

The aim of the current study is to investigate implicit learning and the possible (compensatory) role of explicit learning in children with ASD and SLI, by behavioral (RTs) and ERP measures from an SRT task. Based on previous literature, we expected that TD children would tend to use mainly automatic processes during sequence learning, and that this would be reflected by an N2b enhancement, whereas autistic and SLI children would show more intentional or top‐down learning, reflected by a P3 enhancement. We hypothesized that these different learning styles would lead to intact behavioral performance (measured in RTs) in ASD, but to impaired behavioral performance in SLI on a probabilistic part of the task, for which we assumed implicit processes to be dominant. Furthermore, we expected similar behavioral performance for ASD, SLI, and TD on a deterministic part of the task, for which we assumed that both incidental and intentional processes can be effective. In addition, we explored how these different learning strategies affect explicit knowledge as measured by verbal reports and RT‐drops. Differentiating developmental communication disorders (e.g., in ASD and SLI) in terms of cognitive processes, such as learning processes, is necessary for tailored assessment and treatment [Vissers & Koolen, 2016].

## Methods

### Participants

Sixteen children with an ASD diagnosis, 13 children with an SLI diagnosis, and 17 typically developing (TD) children without a history of psychiatric disorder were recruited (see Supporting Information Appendix 1 for details on drop‐out). The three groups were matched on age, IQ and sex (see Table 1 for details). ASD and SLI diagnoses were made by a clinical psychologist or psychiatrist. Most SLI children (11/13) were diagnosed by a multidisciplinary team from Dutch centers specialized at communication disorders (i.e., Royal Dutch Kentalis). Data from one autistic girl and one boy with SLI could only be used for the behavioral and EEG‐analyses regarding the probabilistic condition due to logistic reasons. All children were free of major neurological disorders and had (corrected‐to) normal vision. Informed consent was obtained from the parents before participating.

**Table 1:** Groups’ Demographic Characteristics

	TD group	ASD group	SLI group	*P*‐value^a^	Post hoc
	(*n* = 17)	(*n* = 16)	(*n* = 13)		
	*M (SD)*	*M (SD)*	*M (SD)*		
Age (years)	11.2 (0.76)	11.3 (0.93)	11.3 (0.64)	0.83	–
Sex (F:M)	9:8	7:9	4:9	0.48	–
IQ^b^	105.4 (13.4)	96.6 (13.5)	99.9 (10.4)	0.15	–
SRS^c^	47.3 (9.6)	67.7 (11.5)	70.0 (18.6)	<0.001	TD<ASD = SLI

a *P*‐value of statistical tests comparing ASD, SLI, and TD group (i.e., one‐way ANOVA's with Group (TD, ASD, SLI) as between‐subject factor for age, IQ and SRS; and a Chi‐square test for sex).

b Based on subtests matrices and spatial span of the Wechsler Nonverbal Scale of Ability (WNV).

c Social Responsiveness Scale (SRS) was missing from 5 TD, 2 ASD, and 3 SLI children.

### Procedures

#### General procedures

The children were tested at their schools or at their usual clinical practice in two short sessions or one longer session. The abbreviated Wechsler Nonverbal Scale of Ability [WNV; Wechsler & Naglieri, 2006] was administered to estimate IQ. The parents were asked to complete the Social Responsiveness Scale [SRS; Constantino & Todd, 2005], a questionnaire consisting of 65 items with 4‐point Likert‐scale answer options, which measures the children's levels of social impairments related to ASD.

#### SRT task

**Figure 1:**
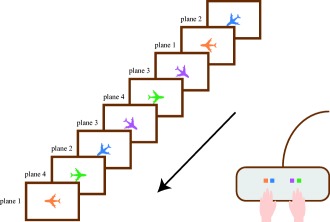
In the current SRT task, the child is asked to give a motor response to a picture of a plane pointing in one of four directions. Unknown to the child, the direction of the arrow is determined by a repeating 8‐element sequence (i.e., 2‐1‐3‐4‐3‐2‐4‐1).

A Dell Latitude 5,450 laptop was used for the SRT task. The task stimuli were centered in order to reduce eye movements in EEG recordings. The children were asked to respond as fast as possible by button press to a picture of a plane pointing in one of four directions; each direction was mapped onto one of four colored response keys on a Logitech G510s keyboard (Fig. 1). Unknown to the child, the orientations and mapped colors of the planes followed a sequence.

The SRT task consisted of a practice task (48 trials not following a sequence) and five target tasks with short breaks in between, in which two sequences were repeated. The experiment started with 60 repetitions of the first sequence ‘2‐1‐3‐4‐3‐2‐4‐1’ (tasks 1 and 2, and first half of task 3), directly followed by 60 repetitions of the second sequence ‘4‐3‐4‐1‐3‐2‐1‐2’ (second half of task 3, and tasks 4 and 5). Both sequences were second‐order in nature: two subsequent elements uniquely predicted the next element. We ensured both sequences contained (a) no repeating elements; (b) only one ‘serial’ triplet (e.g., 1‐2‐3); and (c) only two ‘alternating’ triplets (e.g., 1‐2‐1). The first sequence was probabilistic in nature, including a ‘deviant’ trial in every sequence repetition at a semi‐randomized position (to minimize its predictability). Deviant trials had a random direction under the constraints that the trial did not repeat the direction of adjacent stimuli and that each stimulus was equally often presented. These randomized positions of the deviant trials were in turn randomized across children. The second sequence was deterministic in nature, that is, not including deviant trials. Response‐to‐stimulus interval was set at 500 ms.

After the experiment, a short verbal interview was administered to assess subjective explicit knowledge of the deterministic sequence. This started with: “Did you notice anything about the experiment?.” If the answer was negative, children were told that planes followed a sequence, and they were asked to describe this sequence and encouraged to guess. They were allowed to point at the buttons instead of describing the sequence in words.

#### EEG recordings

EEG was recorded using 32 active electrodes (10–20 arrangement), referenced online to the left mastoid. Data were referenced offline to both mastoids. To control for horizontal and vertical eye movements, electrooculogram (EOG) was recorded from the outer ocular canthi and the left sub‐ and supraorbital ridges. EEG and EOG signals were sampled at 500 Hz, filtered online between 0.016 Hz and 1000 Hz.

### Statistical Analyses

For all analyses, the alpha level was set at 0.05. Greenhouse Geisser correction was applied where the sphericity assumption was violated. EEG data analyses were performed using the FieldTrip MATLAB toolbox developed at the Donders Institute for Brain, Cognition, and Behavior, Nijmegen [Oostenveld, Fries, Maris, & Schoffelen, 2011; http://www.fieldtriptoolbox.org].

To correct for multiple comparisons when investigating significant interaction effects from the main analyses, a Bonferroni adjusted alpha level of 0.05/8 = 0.00063 was used for the 8‐follow‐up *t*‐tests in the behavioral analyses and an adjusted alpha level of 0.005 (= 0.05/10) was used for the 10‐follow‐up *t*‐tests in the ERP analyses.

#### Data preparation

For the behavioral analyses, the trials of each sequence (i.e., probabilistic and deterministic) were split into 3 blocks of 20 sequences (160 trials) to assess learning over time. Extreme outliers were determined for standard trials as RTs 1.5× Interquartile Range (IQR) +/− the median RT of each block, and for deviant trials as +/−1.5 × IQR over 2 blocks. Trials with erroneous responses and the subsequent trial as well as trials after a deviant trial were removed.

Major EEG artifacts were detected by visual inspection; electrodes with artifacts in more than 20 trials in the probabilistic condition were discarded entirely. Trials that were overlapping due to premature responses were discarded (i.e., if a child responded within 150 ms, the next stimulus would be presented within the P3 time window; mean number of trials removed per child <2). Subsequently, eye movement artifacts were removed using independent component analyses (ICA). After ICA, further outliers were removed using a semi‐automatic procedure. On average 149 (out of 210) standard trials and 24 (out of 30) deviant trials were included in each half of the probabilistic condition for ERP analyses. The groups did not differ in number of included trials (*P* = 0.87) or channels (*P* = 0.52).

#### Behavioral probabilistic and deterministic learning

Behavioral learning was investigated with a Group (ASD, SLI, TD) × Trial Type (Standard, Deviant) × Block (3) repeated measures ANOVA with mean RT as dependent variable for the probabilistic condition, and a Group (ASD, SLI, TD) × Block (3) ANOVA for the deterministic condition. Although not the focus of the current paper, similar ANOVAs were conducted on mean number of errors, in which the number of errors on standard trials in the probabilistic condition was divided by seven, because there were seven times more standard trials than deviant trials.

#### Explicit knowledge: RT‐drops and verbal reports

Explicit knowledge was assessed by number of RT‐drops [similar statistical procedures as in Wessel, Haider, & Rose, 2012]. Investigating awareness using RT data is premised on the notion that as soon as a participant develops awareness of learned information, RTs will drop steeply and abruptly (i.e., RT‐drop), which is not seen in implicit knowledge. Learning a sequential structure is a gradual process, in which elements rather than the whole sequence are learned [e.g., Schlaghecken et al., 2000]. In the current task, the sequence can be split into eight elements or triplets. RT‐drops can be determined by comparing these eight triplets. For each of the eight triplets, we first concatenated the RTs of all their member trials into a single pseudo time series, which was subsequently smoothed by applying a median filter of lag 3. Afterward, this time series was made monotonically decreasing by replacing every RT by the minimum of itself and all preceding RTs, starting from the first RT. These new pseudo time series of each of the eight triplets were then aligned for the purpose of finding RT‐drops. RT‐drops occurred when (a) the lowest RT (of eight RTs) at each aligned pseudo time point was smaller than the 99% confidence interval (99% CI) calculated using the median and standard deviation of the other RTs, and (b) when the subsequent two RTs of the pseudo time series of this triplet were below the upper bound of the CI of the current RT (using a standard deviation of the median filtered data of that triplet.

The verbal reports of the deterministic condition were rated based on the number of recalled triplets. Because many children reported incorrect elements too, a verbal score was computed by multiplying the number of recalled triplets by the number of correct triplets divided by the total number of recalled triplets. For example, if a child reported two correct triplets, and three incorrect triplets, it's score was 2 × 2/5 = 0.80.

Between group (TD, ASD, SLI) differences in number of RT drops and the verbal score were investigated by Kruskal–Wallis H tests.

Spearman correlations were used to explore how explicit knowledge measured in number of RT drops and verbal reports contributed to overall learning performance in the deterministic condition.

#### ERPs

A baseline of −100 ms up to stimulus presentation was used for ERPs. ERP enhancements for deviant trials compared to standard trials were investigated over two halves of the probabilistic condition. No ERPs were analyzed for the deterministic condition because this condition did not include deviant trials.

Based on our previous findings in adults, we were mainly interested in N2b and P3. In our analysis of the children's data we took into account the developmental differences in ERP morphology, amplitudes, and latencies that have been reported previously [e.g., Johnstone, Barry, Anderson, & Coyle, 1996; van Dinteren, Arns, Jongsma, & Kessels, 2014]. Based on findings of a general decrease in latencies with age [e.g., Jost, Conway, Purdy, Walk, & Hendricks, 2015; Ridderinkhof & van der Stelt, 2000], we selected later time windows compared to our adult study: N2b: 350–500 ms post stimulus, and P3: 450–650 ms. A peak search in these time windows was done for each child individually (mean amplitude ±20 ms around the peak) for Fz, Cz, and Pz electrodes separately, with the exception for the N2b in Fz for which a broad negativity without a clear peak was found [see Fig. 3; similar to findings in children from Johnstone et al., 1996], and we therefore used a mean amplitude over the time window (350–500 ms) for analyses.

To explore between group differences, Group (ASD, SLI, TD) × Trial Type (Standard, Deviant) × Half (First Half, Second Half) ANOVAs were conducted.

Because of our within‐group hypotheses we have focused on within‐group Electrode (Fz, Cz, Pz) × Trial Type (Standard, Deviant) × Half (First Half, Second Half) ANOVAs. The effect of interest was a Trial Type effect, which would reflect an ERP enhancement for deviant compared to standard trials. In order to reduce the number of statistical tests, and hence the probability of a Type I error, we only conducted additional *t*‐tests for (interaction) effects of interest, that is, not for Electrode or Half, which would only reflect an overall amplitude change over location (Electrode) or time (Half), irrespective of learning.

## Results

### Behavioral Results

#### Probabilistic learning

**Figure 2:**
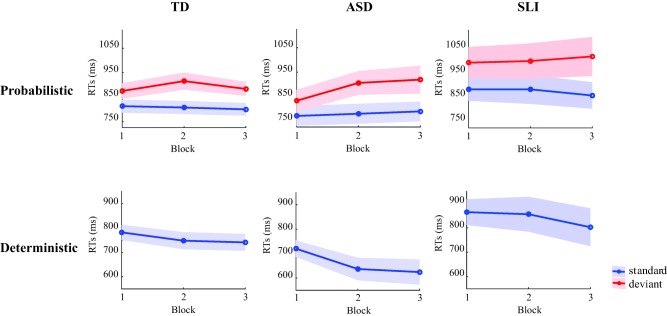
RT performance over blocks in TD (left) ASD (middle) and SLI (right) children, for the probabilistic (upper) and deterministic condition (lower); blue and red lines represent standard and deviant trials, respectively (shaded areas are standard error of the means; SEMs).

A Group (TD, ASD, SLI) × Trial Type (Standard, Deviant) × Block (3) ANOVA revealed a main Trial Type effect, *P* < 0.001, with slower responses to deviant trials (*M* = 927 ms) compared to standard trials (*M* = 819 ms), reflecting sequence learning (see Fig. 2; see Supporting information Appendix 2 for statistical details of behavioral analyses). A main Block effect at trend level was found, *P* = 0.061. A significant Trial Type × Block interaction, *P* = 0.015, with follow‐up *t*‐tests suggesting an effect of Trial Type present at each Block (all *P*‐value's < 0.00063 Bonferroni adjusted alpha level). No main Group effect was found, *P* = 0.084, suggesting similar response speed across groups. No other significant (interaction) effects were found (*P*‐values ≥ 0.19), suggesting no group difference in probabilistic learning.

An ANOVA on errors revealed a main Trial Type effect (*F*(1,43) = 7.62, *P* = 0.008, partial *η* ^2^ = 0.15), with a higher number of errors for deviant (*M* = 1.51) compared to standard trials (*M* = 1.14). No other (interaction) effects were found (*P*‐values ≥ 0.29).

#### Deterministic learning

A Group (TD, ASD, SLI) × Block (3) ANOVA revealed a main Block effect, *P* < 0.001, following a linear trend, *P* < 0.001, reflecting a decrease in RTs over time and hence deterministic learning. A main Group effect was found, *P* = 0.046, with follow‐up *t*‐tests revealing overall slower responses in SLI (RTs: *M* = 841 ms) compared to ASD (*M* = 661 ms), *P* = 0.029, but this difference lost statistical significance against a Bonferroni‐corrected alpha of 0.00063 (= 0.05/8). No other group differences were found: SLI‐TD: *P* = 0.20; ASD‐TD: *P* = 0.12. No Group × Block interaction was found, *P* = 0.26, suggesting similar learning effects across groups.

Regarding number of errors, no significant (interaction) effects were found (all *P*‐values ≥ 0.61).

#### Explicit knowledge

Explicit knowledge was measured by number of RT‐drops and verbal reports. A Kruskal–Wallis H test with Group (TD, ASD, SLI) as factor and RT‐drops as dependent variable revealed no differences between groups, *P* = 0.96. A group difference at trend level was found for verbal reports, *χ* ^2^(2) = 5.80, *P* = 0.055 (see Supporting Information Tables S3 and S4 in Appendix 3 for details on explicit knowledge measures). Follow‐up Mann–Whitney *U*‐tests showed a difference between ASD and TD group, raw *P* = 0.033, in favor of the ASD group, but this difference lost statistical significance when a Bonferroni adjusted alpha level of 0.00063 was used. No differences between ASD/TD and SLI were found (*P*‐value's ≥ 0.073).

Overall deterministic learning, defined as the mean RTs of the first block minus the mean RTs of the last block, correlated significantly with both verbal reports, *r_s_*(44) = 0.48, *P* = 0.001, and RT drops, *r_s_*(44) = 0.31, *P* = 0.043 (across groups).

### Electrophysological Findings

#### Visual inspection

**Figure 3:**
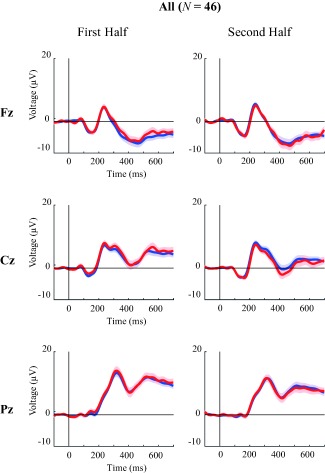
Grand Averages for the first and second half across groups (*N* = 46), for Fz (upper), Cz (middle) and Pz (lower) electrodes, with blue and red lines representing standard and deviant trials respectively (shaded areas are SEMs).

Visual inspection of the grand averages (Fig. 3) shows a broad negativity at Fz, different from the (expected) N2b peak. One potential cause for this relatively unexpected finding could be linked to complex (i.e., diagonal) eye movements despite thorough cleaning of the eye artefacts using ICA (see Methods). However, comparing a subset of children with many eye movements to a subset with only a few eye movements did not substantially change the broad morphology of the ERP at Fz (see Supporing Information Appendix 4). Hence, it seems unlikely that this pattern is driven by eye movements.

**Figure 4:**
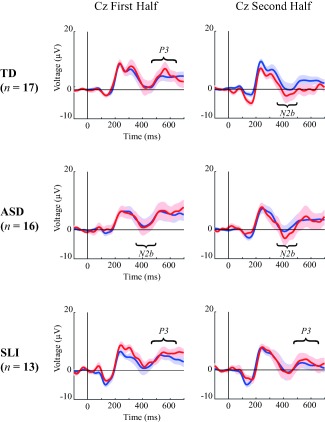
Grand Averages for the first and second half in TD (upper), ASD (middle), and SLI (lower) children, for Cz electrode, with blue and red lines representing standard and deviant trials respectively (shaded areas are SEMs).

**Figure 5:**
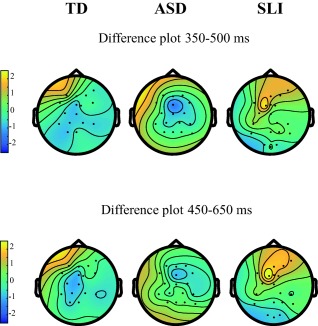
Visualization of the amplitude differences between deviant and standard trials for the N2b time window (upper) and the P3 time window (lower) for the TD (left), ASD (middle), and SLI (right) group. Note the frontal positivity in SLI, compared to the central negativity in TD and ASD.

Visual inspection of the grand averages within groups (Fig. 4) and the scalp distribution (Fig. 5) suggest a central negativity in the TD and ASD group and a fronto‐central positivity in the SLI group.

### Negativity 350–500 ms (fz, cz, pz)

#### Across groups

A Group (TD, ASD, SLI) × Electrode (Fz, Cz, Pz) × Trial Type (Standard, Deviant) × Half (1,2) ANOVA revealed a main Electrode effect, *P* < 0.001, mean amplitudes: Fz: *M* = −5.95 µV; Cz: *M* = −1.67 µV; Pz: *M* = 4.80 µV (see Supporting Information Table S2 for full statistical details on the N2b and P3 across groups analyses). Furthermore, a main Half effect (*P* = 0.002), with lower amplitudes during the second (*M* = −1.75 µV) than during the first Half (*M* = −0.12 µV), and an Electrode × Trial Type interaction, *P* = 0.009. Follow‐up *t*‐tests revealed that the Trial Type effect was only found for the Cz electrode, *P* = 0.005, with more negative amplitudes for deviant (*M* = −2.37 µV) compared to standard trials (*M* = −0.97 µV; other *P*‐values ≥ 0.085). No other statistical significant (interaction) effects were found (*P*‐values ≥ 0.075)

#### Analyses within groups

Figures 4 and 5 show the within‐group Grand Averages at Cz and the ERP scalp distribution respectively.

TD group: a main Electrode effect was found, *P* < 0.001, mean amplitudes: Fz: *M* = −6.91 µV; Cz: *M* = −1.66 µV; Pz: *M* = 6.09 µV (see Supporting Information Table S6 in Appendix 5 for full statistical details on ERP analyses in TD). Furthermore, a main Half effect was found, *P* = 0.025, with lower amplitudes during the second (*M* = −1.59 µV) than during the first Half (*M* = −0.062 µV). A Trial Type × Half interaction effect was also significant *P* = 0.026, with follow‐up analyses revealing a Trial Type only during the second half, *P* = 0.035, reflecting a stronger negativity for deviant (*M* = −2.47 µV) compared to standard trials (*M* = −0.71 µV), but this effect lost significance when Bonferroni‐corrected alpha of 0.005 was applied. No other (interaction) effects were found (*P*‐values ≥ 0.14).

ASD group: a main Electrode effect was found, *P* < 0.001, mean amplitudes: Fz: *M* = −6.84 µV; Cz: *M* = −1.81 µV; Pz: *M* = 4.01 µV (see Supporting Information Table S4 in Appendix 5 for statistical details on ERP analyses in ASD). A main Half effect was found *P* = 0.012, with lower amplitudes during the second (*M* = −2.39 µV) compared to the first Half (*M* = −0.70 µV). Furthermore, a main Trial Type effect, *P* = 0.065, and an Electrode × Trial Type, *P* = 0.052, were found at trend level. Follow‐up *t*‐tests revealed a Trial Type effect at Cz electrode only, *P* < 0.002 (Bonferroni adjusted alpha level: 0.005), with stronger negativity for deviant (*M* = −2.97 µV) compared to standard trials (*M* = −0.64 µV). No other (interaction) effects were found (*P*‐values ≥ 0.47).

SLI group: a main Electrode effect was found, *P* < 0.001, mean amplitudes: Fz: *M* = −3.58 µV; Cz: *M* = −1.51 µV; Pz: *M* = 4.09 µV (see Supporting Information Table S5 in Appendix 5 for statistical details on ERP analyses in SLI). No other (interaction) effects reached statistical significance (*P*‐values ≥ 0.085).

In sum, across groups, an N2b enhancement was found at Cz. Within groups, this effect was present only in ASD, and during the second half in TD, but the latter lost statistical significance when alpha levels were Bonferroni adjusted. No N2b effects were found in SLI.

### P3 450–650 ms (fz, cz, pz)

#### Analyses across groups

A Group (TD, ASD, SLI) × Electrode (Fz, Cz, Pz) × Trial Type (Standard, Deviant) × Half (1,2) ANOVA revealed a main Electrode effect: *P* < 0.001, mean amplitudes: Fz: *M* = −1.40 µV; Cz: *M* = −6.63 µV; Pz: *M* = 12.59 µV. Furthermore, a main Trial Type effect was found, *P* = 0.005, with higher amplitudes for deviant (*M* = 6.57 µV) compared to standard trials (*M* = 5.31 µV), reflecting P3 enhancement. A main Half effect, *P* < 0.001, with higher amplitudes during the first (*M* = 7.04 µV) than during the second Half (*M* = 4.84 µV) was also found. No other (interaction) effects were found (*P*‐values ≥ 0.075)

#### Analyses within groups

TD group: a main Electrode effect was found *P* < 0.001 mean amplitudes: Fz: *M* = −2.68 µV; Cz: *M* = 5.75 µV; Pz: *M* = 12.8 µV. A main Half effect, *P* = 0.008, with higher overall amplitudes during the first (*M* = 6.37 µV) compared to the second Half (*M* = 4.19 µV), and a Trial Type × Half interaction, *P* = 0.034, were found. Follow‐up *t*‐tests revealed a Trial Type only during the first half, *P* = 0.013 (Bonferroni adjusted alpha‐level: 0.005), reflecting a stronger positivity for deviant (*M* = 6.76 µV) compared to standard trials (*M* = 5.49 µV). No other (interaction) effects were found (*P*‐values ≥ 0.19).

ASD group: a main Electrode effect was found, *P* < 0.001, mean amplitudes: Fz: *M* = −1.61 µV; Cz: *M* = 7.35 µV; Pz: *M* = 12.8 µV. Furthermore, a main Half effect was found, *P* = 0.007, with higher amplitudes during the first Half (*M* = 7.17 µV) compared to the second Half (*M* = 5.19 µV). No other (interaction) effects were found (*P*‐values ≥ 0.24).

SLI group: a main Electrode effect was found *P* < 0.001, mean amplitudes: Fz: *M* = 0.53 µV; Cz: *M* = 6.90 µV; Pz: *M* = 12.1 µV. Furthermore, a main Trial Type effect was found, *P* = 0.031, with stronger positivity for deviant (*M* = 7.60 µV) compared to standard trials (*M* = 5.41 µV), reflecting a P3 enhancement. No other (interaction) effects were found (*P*‐values ≥ 0.11).

Thus, across groups, a P3 enhancement was found. Within groups, this effect was found in SLI, and during the first half in TD. No P3 effects were found in ASD.

## Discussion

### Aim

The aim of the current paper was to investigate implicit learning in ASD and SLI using both behavioral and ERP measures on the SRT task, and to explore the potential role of a compensatory explicit learning system in both disorders.

### Implicit Learning in ASD

The autistic children showed similar behavioral learning as TD children, in line with the majority of findings in ASD [Foti et al., 2015; Zwart, Vissers, Kessels, et al., 2017]. Scores on the post‐experimental interviews indicate that autistic children may have more verbal explicit knowledge than TD children, although this difference was only weak. Furthermore, no group differences were found in the RT‐drop measures of explicit knowledge.

Electrophysiologically, probabilistic learning in autistic children was reflected by a central negativity (350–500 ms) enhancement, most likely reflecting an N2b component. Within‐group analyses showed that while this negativity was present during both early and late learning stages in ASD, it was only present during late learning in TD, and not at all in SLI children. An N2b effect has been associated with implicit learning processes on the SRT task [e.g., Fu et al., 2013], although others have found an N2b for both implicit and explicit learners and defined it more broadly as an error monitoring process [Ferdinand et al., 2008]. These findings suggest that autistic children use automatic processes to detect deviants in regularities, and this may be interpreted as a reflection of intact implicit learning.

This strong negativity effect in autistic children contrasts our previous findings in adults, in which we found probabilistic learning to be characterized by an N2b in TD, and rather by a P3 in ASD [Zwart, Vissers, van der Meij, et al., 2017]. Taken together, these findings suggest that autistic adults rely more on intentional or effortful learning strategies (as suggested by the P3), while autistic children show signs of more automatic processes similar to TD children (as suggested by the N2b). It is believed that the declarative (explicit) learning mechanism starts to become more prominent after the age of 12 years [e.g., Janacsek et al., 2012]. Perhaps, the suggested compensatory role of intentional learning in ASD [e.g., Klinger et al., 2007] only becomes prominent in adolescence.

### Implicit Learning in SLI

The SLI children showed similar behavioral learning as the TD children, which was unexpected based on the majority of literature [Obeid et al., 2016; Zwart, Vissers, Kessels, et al., 2017]. However, some studies report a deficit only in retention of learning, not in initial sequence learning [e.g., Desmottes, Maillart, & Meulemans, 2017; Hedenius et al., 2011]. Furthermore, a substantial number of other studies have also reported comparable learning in SLI on the SRT task [Gabriel et al., 2011; Gabriel, Meulemans, Parisse, & Maillart, 2015; Gabriel, Stefaniak, Maillart, Schmitz, & Meulemans, 2012; Lum & Bleses, 2012]. The current findings also suggest that overall responses might be slower is SLI compared to ASD, and variance in the response times seems to be high. These findings may be related to overall motor problems in SLI, but it is important to note that motor deficits could not have adversely affected behavioral and ERP findings of learning, as the learning effects concerned within‐subject factors (i.e., Trial Type effect and/or Block effect). No differences between SLI and TD in explicit knowledge as measured by verbal reports or RT‐drops were found.

Contrary to the ASD results, probabilistic learning in SLI was not reflected by an early negativity, but by a fronto‐central positivity enhancement (450–650 ms), most likely reflecting a P3‐like component. Although overall this effect did not differ from the findings in the other groups, within‐group analyses showed that while this positivity enhancement was present during both early and late stages of learning in SLI, it was only present during early learning in TD, and not at all in ASD. As discussed earlier, the P3 is associated with updating working memory [e.g., Linden, 2005], and on the SRT task with intentional learning [e.g., Ferdinand et al., 2008]. The fronto‐central positivity finding is likely to reflect more controlled processes compared to the earlier negativity found in ASD, and could therefore be in line with the idea of a compensatory role of intentional learning in SLI [Lum et al., 2014]. Thus, it seems that the children with SLI use more effortful strategies and that these strategies are needed throughout the task.

The current ERP findings in SLI also fit in the theoretical framework of an implicit learning deficit in SLI developed by Ullman and Pierpont [2005]. Ullman described that language learning involves both procedural (or implicit) memory involving frontal/basal ganglia circuits, particularly the nigro‐striatal system, and declarative (or explicit) memory mainly relying on hippocampal structures [Ullman, 2004]. Based on findings of a Go‐NoGo study including patients with basal ganglia disorders, it has been suggested that the N2 is associated with the nigo‐striatal system for pre‐motor inhibition, whereas the P3 is more associated with the mesocortico‐limbic system involved in outcome monitoring [Beste, Willemssen, Saft, & Falkenstein, 2010]. In line with this, intracranial findings suggest that hippocampal‐frontal networks are involved in a frontal P3 component [Knight, 1996]. Thus, it could be speculated that our ERP findings in SLI reflect impaired basal ganglia learning (reflected by the absence of the N2b) which is compensated for by hippocampal learning (reflected by the frontal P3) in line with the procedural learning deficit from Ullman and Pierpont [2005].

### Limitations and Future Research

The small sample sizes of the current study might have contributed to the lack of overall group differences in ERP findings even though the within‐group analyses suggested different patterns. The (particularly) small size of the SLI group might have contributed to no statistically significant difference in behavioral learning compared to TD. Alternatively, or in addition, our behavioral analyses with relatively large blocks of trials might not have been sensitive enough to pick up subtle deficits in learning, such as a slower rate of learning. Investigating smaller blocks of trials could reveal if children with SLI need more blocks of training before learning occurs.

Identifying the ERP components N2b and P3 in the current data proved to be challenging, as the number of previous studies examining these components in school‐aged children is very limited. We have chosen later time windows for our ERP search in children compared to our previous adult study, based on general findings of a decrease in ERP latencies with age [e.g., Fuchigami et al., 1993; Johnstone et al., 1996; Polich, Ladish, & Burns, 1990; Ridderinkhof & van der Stelt, 2000; van Dinteren et al., 2014; but also see Tomé, Barbosa, Nowak, & Marques‐Teixeira, 2015]. It could be argued that the current findings reflect later ERP components than the N2b and P3, which might also partially explain the broad frontal negativity in our findings (rather than the peak‐like N2b). For example, our negativity findings may be interpreted as an N400, which has been associated to meaning processes in verbal and nonverbal language studies [see Kutas & Federmeier, 2011]. The N400 is thought to reflect processes relatively outside awareness [e.g., Curran & Cleary, 2003; Kutas & Federmeier, 2011], although the modulating role of selective attention in the N400 suggest that it is not fully automatic [Kutas & Federmeier, 2011]. Because of the nature of our task (i.e., a sequence of monotomous stimuli) and the previous findings of N2b and P3 components on similar tasks [e.g., Ferdinand et al., 2008; Jost, Conway, Purdy, & Hendricks, 2011; Zwart, Vissers, van der Meij, et al., 2017], our findings most likely reflect an N2b and P3, but it is important to note that questioning the terminology of the ERP findings would not change the overall interpretation of an earlier negativity as a reflection of a largely automatic detection of deviants, and a later positivity as a reflection of more controlled processes.

The ambiguity in identifying ERP components in children is not unique to our study, and the field of developmental changes in ERPs could greatly benefit from future research on age related changes in ERP characteristics. Currently, it is questionable whether the few individual child studies have identified the same ERPs. For example, one child study using a statistical learning paradigm identified a P3 peak in a time window of 190–350 ms [Jeste et al., 2015], while another study using a similar paradigm investigated a P3 peak between 400 and 700 ms [Jost et al., 2015]. Hence, in order to reach consensus, studies including different age groups investigating popular ERPs (such as the N2b and the P3) on the same paradigm are needed.

### Conclusions and Clinical Implications

The current study shows that implicit statistical learning is intact in autistic children, both in behavioral as well as in electrophysiological respect. The potential overreliance on explicit strategies might still play a role later in development during adolescence, when intentional learning mechanisms start to become more prominent. However, at least for children with ASD, interventions should not solely rely on intentional learning. A more implicit way of teaching a skill can be found in errorless learning techniques, in which the skill is trained while minimalizing error making [e.g., Terrace, 1963]. In ASD, this approach has been successfully applied to improve social engagement, in which a desired social interaction was created followed by a gradual removal of prompts from the situation [Stevenson, Krantz, & McClannahan, 2000].

In contrast, learning seems different for children with SLI, as our electrophysiological findings suggest that these children use more controlled, effortful strategies to reach the same behavioral performance. This might be due to the use of intentional learning to compensate for an implicit learning deficit [Ullman & Pierpont, 2005]. Interventions for children with SLI could aim at compensating for the limited implicit learning abilities, by offering more explicit training in learning statistical regularities, such as grammar and social situations.

## Supporting information

Additional Supporting Information may be found in the online version of this article at the publisher's website.

Supporting Information Table 2

Supporting Information Table 3

Supporting Information Table 4

Supporting Information Table 5

Supporting Information Table 6

Supporting Information Table 7

Supporting Information Table 8
