# Supplementary material for: Implicit learning seems to come naturally for children with autism, but not for children with specific language impairment: Evidence from behavioral and ERP data
Source: Autism Res. 2018 Apr 20;11(7):1050–61. doi: 10.1002/aur.1954 (PMC6120494; doi:10.1002/aur.1954)
Supplement: Supplementary file 1 — Supporting Information Table 2 [file AUR-11-1050-s001.docx]

**Table 2**

*Behavioral analyses on RTs for the probabilistic and the deterministic condition (N = 46)*

| Task condition | Effect | df1 | df2 | *F/t* | *P* | partial *ƞ*² |
| --- | --- | --- | --- | --- | --- | --- |
| Probabilistic | Group | 2 | 43 | 2.62 | .084 | .11 |
|  | Trial Type | 1 | 43 | 70.3 | <.001** | .62 |
|  | Trial Type * Group | 2 | 43 | 1.75 | .19 | .075 |
|  | Block | 1.70 | 73.2 | 3.05 | .061 | .066 |
|  | Block * Group | 3.40 | 73.2 | .94 | .43 | .042 |
|  | Trial Type * Block | 2 | 86 | 4.40 | .015* | .093 |
|  | *Block 1: Trial Type* | *-* | *45* | *5.76* | *<.001*** | *-* |
|  | *Block 2: Trial Type* | *-* | *45* | *6.21* | *<.001*** | *-* |
|  | *Block 3: Trial Type* | *-* | *45* | *6.63* | *<.001*** | *-* |
|  | Trial Type * Block * Group | 4 | 86 | .78 | .54 | .035 |
| Deterministic | Group | 2 | 41 | 3.33 | .046* | .140 |
|  | *TD vs. ASD* | *-* | *30* | *1.59* | *.12* | *-* |
|  | *TD vs. SLI* | *-* | *16.6* | *1.22* | *.24* | *-* |
|  | *ASD vs. SLI* | *-* | *25* | *2.31* | *.029** | *-* |
|  | Block | 1.55 | 63.7 | 12.6 | <.001** | .24 |
|  | *Following a linear trend* | *1* | *41* | *16.1* | *<.001*** | *.28* |
|  | Block * Group | 3.11 | 63.7 | 1.37 | .26 | .063 |

*** p*-value < .05*

**** p*-value < .001*
