# Supplementary material for: Implicit learning seems to come naturally for children with autism, but not for children with specific language impairment: Evidence from behavioral and ERP data
Source: Autism Res. 2018 Apr 20;11(7):1050–61. doi: 10.1002/aur.1954 (PMC6120494; doi:10.1002/aur.1954)
Supplement: Supplementary file 2 — Supporting Information Table 3 [file AUR-11-1050-s002.docx]

**Table 3**

*Explicit knowledge: verbal reports*

|  | **TD group** | **ASD group** | **SLI group** | **Total** |
| --- | --- | --- | --- | --- |
|  | **(*n* = 17)** | **(*n* = 16)** | **(*n* = 13)** | **(*N* = 46)** |
| Non-verbalizers (0 triplets) | 7 | 2 | 3 | 12 |
| Partial Verbalizers (1-7 triplets) | 8 | 7 | 5 | 20 |
| Full Verbalizers (8 triplets) | 2 | 6 | 4 | 12 |
| Missing | - | 1 | 1 | 2 |
| Total | 17 | 16 | 13 | 46 |
