# Supplementary material for: Implicit learning seems to come naturally for children with autism, but not for children with specific language impairment: Evidence from behavioral and ERP data
Source: Autism Res. 2018 Apr 20;11(7):1050–61. doi: 10.1002/aur.1954 (PMC6120494; doi:10.1002/aur.1954)
Supplement: Supplementary file 4 — Supporting Information Table 5 [file AUR-11-1050-s004.docx]

**Table 5**

*Overall ERP effects (N = 46)*

| ERP | Effect | df1 | df2 | *F/t* | *p* | partial *ƞ*² |
| --- | --- | --- | --- | --- | --- | --- |
| N2b | Group | 2 | 43 | .085 | .92 | .004 |
|  | Electrode | 1.40 | 60.2 | 94.0 | <.001** | .69 |
|  | Electrode * Group | 2.80 | 60.2 | 2.12 | .11 | .090 |
|  | Trial Type | 1 | 43 | 2.01 | .16 | .045 |
|  | Trial Type * Group | 2 | 43 | 1.17 | .32 | .052 |
|  | Half | 1 | 43 | 11.4 | .002* | .21 |
|  | Half * Group | 2 | 43 | .014 | .97 | .001 |
|  | Electrode * Trial Type | 1.39 | 59.8 | 6.13 | .009* | .13 |
|  | *Fz: Trial Type* | *-* | *45* | *-.47* | *.64* | *-* |
|  | *Cz: Trial Type* | *-* | *45* | *2.96* | *.002** | *-* |
|  | *Pz: Trial Type* | *-* | *45* | *1.64* | *.11* | *-* |
|  | Electrode * Trial Type * Group | 2.78 | 59.8 | .83 | .47 | .037 |
|  | Trial Type * Half | 1 | 43 | 3.33 | .075 | .072 |
|  | Trial Type * Half * Group | 2 | 43 | .24 | .79 | .011 |
|  | Electrode * Trial Type * Half | 1.35 | 58.2 | .19 | .83 | .004 |
|  | Electrode * Trial Type * Half * Group | 2.71 | 58.2 | .19 | .88 | .009 |
| P3 | Group | 2 | 43 | .14 | .87 | .006 |
|  | Electrode | 2 | 57.9 | 166.4 | <.001** | .80 |
|  | Electrode * Group | 4 | 57.9 | 1.27 | .29 | .056 |
|  | Trial Type | 1 | 43 | 8.94 | .005* | .17 |
|  | Trial Type * Group | 2 | 43 | .93 | .40 | .041 |
|  | Half | 1 | 43 | 16.9 | <.001** | .28 |
|  | Half * Group | 2 | 43 | .067 | .94 | .003 |
|  | Electrode * Trial Type | 1.33 | 57.5 | .064 | .87 | .001 |
|  | Electrode * Trial Type * Group | 2.68 | 57.5 | 1.16 | .33 | .051 |
|  | Trial Type * Half | 1 | 43 | 3.30 | .076 | .071 |
|  | Trial Type * Half * Group | 2 | 43 | .38 | .69 | .017 |
|  | Electrode * Trial Type * Half | 1.45 | 62.5 | .041 | .92 | .001 |
|  | Electrode * Trial Type * Half * Group | 2.91 | 62.5 | .44 | .72 | .020 |

*Note: raw* p*-values are reported, but additional* t-*tests to investigate interaction effects have been corrected*

*** p*-value < .05*

**** p*-value < .001*
