# Supplementary material for: Implicit learning seems to come naturally for children with autism, but not for children with specific language impairment: Evidence from behavioral and ERP data
Source: Autism Res. 2018 Apr 20;11(7):1050–61. doi: 10.1002/aur.1954 (PMC6120494; doi:10.1002/aur.1954)
Supplement: Supplementary file 5 — Supporting Information Table 6 [file AUR-11-1050-s005.docx]

**Table 6**

*ERP effects in TD (n = 17)*

| ERP | Effect | df1 | df2 | *F/t* | *p* | partial *ƞ*² |
| --- | --- | --- | --- | --- | --- | --- |
| N2b | Electrode | 1.32 | 21.2 | 47.1 | <.001** | .75 |
|  | Trial Type | 1 | 16 | 2.42 | .14 | .13 |
|  | Half | 1 | 16 | 6.08 | .025* | .28 |
|  | Electrode * Trial Type | 1.28 | 20.5 | 1.57 | .23 | .090 |
|  | Trial Type * Half | 1 | 16 | 6.02 | .026* | .27 |
|  | *1^st^ half: Trial Type* | *1* | *16* | *.030* | *.86* | *.002* |
|  | *2^nd^ half: Trial Type* | *1* | *16* | *5.31* | *.035** | *.25* |
|  | Electrode * Trial Type * Half | 1.33 | 21.3 | .16 | .77 | .010 |
| P3 | Electrode | 1.29 | 20.6 | 70.5 | <.001** | .82 |
|  | Trial Type | 1 | 16 | 1.86 | .19 | .10 |
|  | Half | 1 | 16 | 9.01 | .008* | .36 |
|  | Electrode * Trial Type | 1.25 | 20.0 | .10 | .90 | .006 |
|  | Trial Type * Half | 1 | 16 | 5.35 | .034* | .25 |
|  | *1^st^ half: Trial Type* | *-* | *16* | *2.79* | *.013** | *-* |
|  | *2^nd^ half: Trial Type* | *-* | *16* | *.72* | *.49* | *-* |
|  | Electrode * Trial Type * Half | 2 | 32 | .74 | .49 | .044 |

*** p*-value < .05*

**** p*-value < .001*
