# Supplementary material for: Implicit learning seems to come naturally for children with autism, but not for children with specific language impairment: Evidence from behavioral and ERP data
Source: Autism Res. 2018 Apr 20;11(7):1050–61. doi: 10.1002/aur.1954 (PMC6120494; doi:10.1002/aur.1954)
Supplement: Supplementary file 6 — Supporting Information Table 7 [file AUR-11-1050-s006.docx]

**Table 7**

*ERP effects in ASD (n = 16)*

| ERP | Effect | df1 | df2 | *F/t* | *p* | partial *ƞ*² |
| --- | --- | --- | --- | --- | --- | --- |
| N2b | Electrode | 1.41 | 21.2 | 29.36 | <.001** | .66 |
|  | Trial Type | 1 | 15 | 3.97 | .065 | .21 |
|  | Half | 1 | 15 | 8.07 | .012* | .35 |
|  | Electrode * Trial Type | 1.39 | 20.8 | 3.82 | .052 | .20 |
|  | *Fz: Trial Type* | *-* | *15* | *.70* | *.50* | *-* |
|  | *Cz: Trial Type* | *-* | *15* | *3.64* | *.002** | *-* |
|  | *Pz: Trial Type* | *-* | *15* | *.74* | *.47* | *-* |
|  | Trial Type * Half | 1 | 15 | .75 | .40 | .047 |
|  | Electrode * Trial Type * Half | 1.26 | 18.9 | .20 | .72 | .013 |
| P3 | Electrode | 1.47 | 22.1 | 53.3 | <.001** | .78 |
|  | Trial Type | 1 | 15 | 1.52 | .24 | .092 |
|  | Half | 1 | 15 | 9.92 | .007* | .40 |
|  | Electrode * Trial Type | 2 | 30 | 1.09 | .35 | .068 |
|  | Trial Type * Half | 1 | 15 | .23 | .64 | .015 |
|  | Electrode * Trial Type * Half | 2 | 30 | .25 | .78 | .016 |

*** p*-value < .05*

**** p*-value < .001*
