# Supplementary material for: Implicit learning seems to come naturally for children with autism, but not for children with specific language impairment: Evidence from behavioral and ERP data
Source: Autism Res. 2018 Apr 20;11(7):1050–61. doi: 10.1002/aur.1954 (PMC6120494; doi:10.1002/aur.1954)
Supplement: Supplementary file 7 — Supporting Information Table 8 [file AUR-11-1050-s007.docx]

**Table 8**

*ERP effects in SLI (n = 13)*

| ERP | Effect | df1 | df2 | *F/t* | *p* | partial *ƞ*² |
| --- | --- | --- | --- | --- | --- | --- |
| N2b | Electrode | 1.32 | 15.9 | 26.2 | < .001** | .69 |
|  | Trial Type | 1 | 12 | .12 | .74 | .010 |
|  | Half | 1 | 12 | 1.66 | .22 | .12 |
|  | Electrode * Trial Type | 2 | 24 | 2.74 | .085 | .19 |
|  | Trial Type * Half | 1 | 12 | .26 | .62 | .021 |
|  | Electrode * Trial Type * Half | 1.40 | 16.8 | .23 | .72 | .019 |
| P3 | Electrode | 1.16 | 13.87 | 55.6 | <.001** | .82 |
|  | Trial Type | 1 | 12 | 6.01 | .031* | .33 |
|  | Half | 1 | 12 | 2.93 | .11 | .20 |
|  | Electrode * Trial Type | 1.32 | 15.8 | 1.24 | .30 | .094 |
|  | Trial Type * Half | 1 | 12 | .98 | .34 | .075 |
|  | Electrode * Trial Type * Half | 1.10 | 13.2 | .029 | .89 | .002 |

*** p*-value < .05*

**** p*-value < .001*
